# A Tale of Two Viruses: Coinfections of Monkeypox and Varicella Zoster Virus in the Democratic Republic of Congo

**DOI:** 10.4269/ajtmh.20-0589

**Published:** 2020-12-07

**Authors:** Christine M. Hughes, Lindy Liu, Whitni B. Davidson, Kay W. Radford, Kimberly Wilkins, Benjamin Monroe, Maureen G. Metcalfe, Toutou Likafi, Robert Shongo Lushima, Joelle Kabamba, Beatrice Nguete, Jean Malekani, Elisabeth Pukuta, Stomy Karhemere, Jean-Jacques Muyembe Tamfum, Emile Okitolonda Wemakoy, Mary G. Reynolds, D. Scott Schmid, Andrea M. McCollum

**Affiliations:** 1 Poxvirus and Rabies Branch, Division of High-Consequence Pathogens and Pathology, National Center for Emerging and Zoonotic Infectious Diseases, U.S. Centers for Disease Control and Prevention, Atlanta, Georgia;; 2 Bacterial Special Pathogens Branch, Division of High-Consequence Pathogens and Pathology, National Center for Emerging and Zoonotic Infectious Diseases, U.S. Centers for Disease Control and Prevention, Atlanta, Georgia;; 3 Infectious Diseases Pathology Branch, Division of High-Consequence Pathogens and Pathology, National Center for Emerging and Zoonotic Infectious Diseases, U.S. Centers for Disease Control and Prevention, Atlanta, Georgia;; 4 Viral Vaccine Preventable Diseases Branch, Division of Viral Diseases, National Center for Immunizations and Respiratory Diseases, U.S. Centers for Disease Control and Prevention, Atlanta, Georgia;; 5 Kinshasa School of Public Health, Kinshasa, Democratic Republic of Congo;; 6 Ministry of Health, Kinshasa, Democratic Republic of Congo;; 7 U.S. Centers for Disease Control and Prevention, Kinshasa, Democratic Republic of Congo;; 8 Department of Biology, University of Kinshasa, Kinshasa, Democratic Republic of Congo;; 9 Institut National de Recherche Biomédicale, Kinshasa, Democratic Republic of Congo

## Abstract

Recent enhanced monkeypox (MPX) surveillance in the Democratic Republic of Congo, where MPX is endemic, has uncovered multiple cases of MPX and varicella zoster virus (VZV) coinfections. The purpose of this study was to verify if coinfections occur and to characterize the clinical nature of these cases. Clinical, epidemiological, and laboratory results were used to investigate MPX/VZV coinfections. A coinfection was defined as a patient with at least one *Orthopoxvirus*/MPX-positive sample and at least one VZV-positive sample within the same disease event. Between September 2009 and April 2014, 134 of the 1,107 (12.1%) suspected MPX cases were confirmed as MPX/VZV coinfections. Coinfections were more likely to report symptoms than VZV-alone cases and less likely than MPX-alone cases. Significantly higher lesion counts were observed for coinfection cases than for VZV-alone but less than MPX-alone cases. Discernible differences in symptom and rash severity were detected for coinfection cases compared with those with MPX or VZV alone. Findings indicate infection with both MPX and VZV could modulate infection severity. Collection of multiple lesion samples allows for the opportunity to detect coinfections. As this program continues, it will be important to continue these procedures to assess variations in the proportion of coinfected cases over time.

## INTRODUCTION

Monkeypox virus (MPXV) is an endemic *Orthopoxvirus* (OPXV) in West and Central Africa. Most of the human infections reported each year are from the Congo Basin of the Democratic Republic of Congo (DRC). Monkeypox (MPX) disease presentation, including the characteristic rash, most closely resembles that of smallpox, an eradicated disease caused by a closely related OPXV. Monkeypox disease presentation is often confused with another febrile rash illness, varicella zoster virus (VZV). Previous evaluations of MPX laboratory-based surveillance have noted a large number of VZV infections among cases that were MPX negative, often in the same area where MPX cases occurred.^2–4^

Clinical diagnosis of MPX based on rash examination during the early macule and papule stages can be challenging. Key differences between MPX and VZV disease presentation at symptom onset and during illness progression can help to establish a presumptive diagnosis.^5^ Monkeypox patients often experience a febrile prodrome with high fever 1–4 days before rash onset, whereas a low-grade fever at rash onset is more common for VZV. Lymphadenopathy is also a distinguishing MPX characteristic. Lesions on the palms of the hands and soles of the feet are often noted in MPX patients; although this feature is not recognized as a significant VZV characteristic, it has also been noted in VZV patients.^6^ Monkeypox lesions are firm, deep-seated, well-circumscribed with a central point of umbilication; present at a single stage of development on any one site of the body (e.g., all pustules on the arm); and evolve slowly with each stage, lasting 1–2 days.^5,7,8^ Varicella zoster virus lesions are usually more superficial in appearance with irregular borders, can be present at multiple stages on any one site on the body, and rapidly evolve from macules to crusts within 24 hours. Varicella zoster virus infection results in lifelong latent infection of the dorsal root ganglia, and reactivation with a dermatomally distributed painful rash illness (zoster) is common, particularly in persons older than 60 years.^9^

Epidemiological patterns also differ between MPX and VZV. Monkeypox is a zoonotic disease with relatively limited human-to-human transmission.^10,11^ Varicella zoster virus does not have an animal reservoir and is characterized by widespread human-to-human transmission. Secondary attack rates for VZV among susceptible household contacts were estimated at 87%.^12^ In temperate climates, primary infection of VZV generally occurs in early childhood. By contrast, primary infection of VZV in tropical regions often occurs in adolescents and adults.^13,14^ Seroprevalence rates for VZV are understudied in Africa, and few studies of VZV seroprevalence have been conducted in MPX-endemic countries. In Ghana, 45% (54 of 120) of healthy participants aged 16–46 years were seropositive for VZV.^15^ A serosurvey of children aged 6–59 months in the DRC showed a VZV seroprevalence of 8%.^16^ Patients with MPXV and VZV coinfections have been previously noted in the DRC.^3,17–19^

We describe the detection of both MPXV and VZV in persons investigated for suspected MPX infection via an enhanced MPX surveillance program in Tshuapa Province, DRC. The unexpectedly large number of detected coinfections was examined for biological plausibility. The purpose of this study was to confirm that MPX and VZV coinfections do occur and to characterize the clinical nature of these cases. Case demographics, symptom, and clinical severity, including rash burden, are described and evaluated in comparison to individuals with MPX or VZV infection alone. Temporal and locality trends of coinfection cases were investigated to determine if coinfections are present at a rate greater than would be expected by chance alone. The findings of this analysis will have implications for MPX disease surveillance and case management in the DRC.

## MATERIALS AND METHODS

### Surveillance system.

Human MPX is a mandatory reportable disease in the DRC. An MPX case definition was implemented in October 2010 for enhanced surveillance in Tshuapa Province. A suspected case was defined as follows: a patient with a vesicular pustular eruption characterized by hard and deep pustules and with at least one of the following symptoms: fever preceding the eruption, lymphadenopathy (inguinal, axillary, or cervical), and/or pustules or crusts on the palms of the hands or soles of the feet. A formal investigation of a suspected MPX case included the collection of samples and the completion of a case investigation form by a trained surveillance officer. Surveillance officers are provided with sample collection kits that contain two swabs (Catch-All™ swab, Epicentre, Madison, WI), two sterile tube (Sarstedt 2 ml o-ring tubes, Netwon, NC) for crust collection, two tools to unroof crusts (Qosina, Ronkonkoma, NY), and an MPX-specific case investigation form. Surveillance officers are trained to collect a sample from two different lesions, for a total of at least two samples for each case. Lesion swabs and/or lesion crusts are the preferred samples for MPX diagnostics. Blood samples were rarely, but sometimes, collected.

### Ethical statement.

These activities were determined to not be research by a CDC human subjects advisor.

### Laboratory diagnosis.

One sample type from every case was processed and tested at the Institut National de Recherche Biomédicale (INRB) in Kinshasa. The INRB tested for OPX DNA signatures using an OPX-generic real-time PCR assay.^20^ In the absence of OPX DNA amplification, a second real-time PCR assay for VZV-specific DNA signatures was conducted (U.S. Army Medical Research Institute of Infectious Diseases, unpublished). Patient samples that yielded positive findings for OPX were not tested for VZV at the INRB. Prepared DNA remainders from these samples and unprocessed additional lesion samples were shipped to the CDC Poxvirus Laboratory, Atlanta, GA.

The unprocessed lesion samples received at the Poxvirus Laboratory were processed, and DNA was extracted. The DNA, along with DNA previously processed at the INRB, was tested using the MPXV-specific real-time PCR and VZV-specific real-time PCR assays.^21–23^ Including diagnostic results from both the CDC and INRB, a patient was considered a confirmed MPX case if at least one sample was either positive at the INRB (OPX-specific assay) or positive at CDC’s Poxvirus Laboratory (MPXV-specific real-time PCR). Independent of the laboratory results for MPX, a patient was considered a confirmed VZV case if at least one sample was positive for VZV at the CDC or INRB.

Viral isolation was attempted for lesion samples that yield an MPXV real-time PCR Ct value less than 32. Viral isolation was performed in a T25 flask of African green monkey kidney cells (BSC-40). Roswell Park Memorial Institute (RPMI) 1640 media was supplemented with 2% heat-inactivated fetal bovine serum, L-glutamine, penicillin–streptomycin, amphotericin B, and gentamicin. Inocula comprising 2 mL RPMI 1640 plus 20 μL sample were incubated with the cells from 1 hour to overnight at 35.5°C and 5–6% CO_2_ before adding fresh medium. Flasks were observed daily for 9 days for cytopathic effect. Positive flasks were harvested when the monolayer was approximately 100% infected, or if the cell culture was older than 1 week after initial plating, and cytopathic effect was limited.

Crust samples that were dual positive at the CDC were evaluated by electron microscopy (EM). A small amount of the processed sample (crust homogenate) was mixed 1:1 with 5% paraformaldehyde and allowed to inactivate for 24 hours. Electron microscopy grids were enhanced with 1% Alcian blue and prepared using the drop-to-drop method.^24^

**Figure 1.**
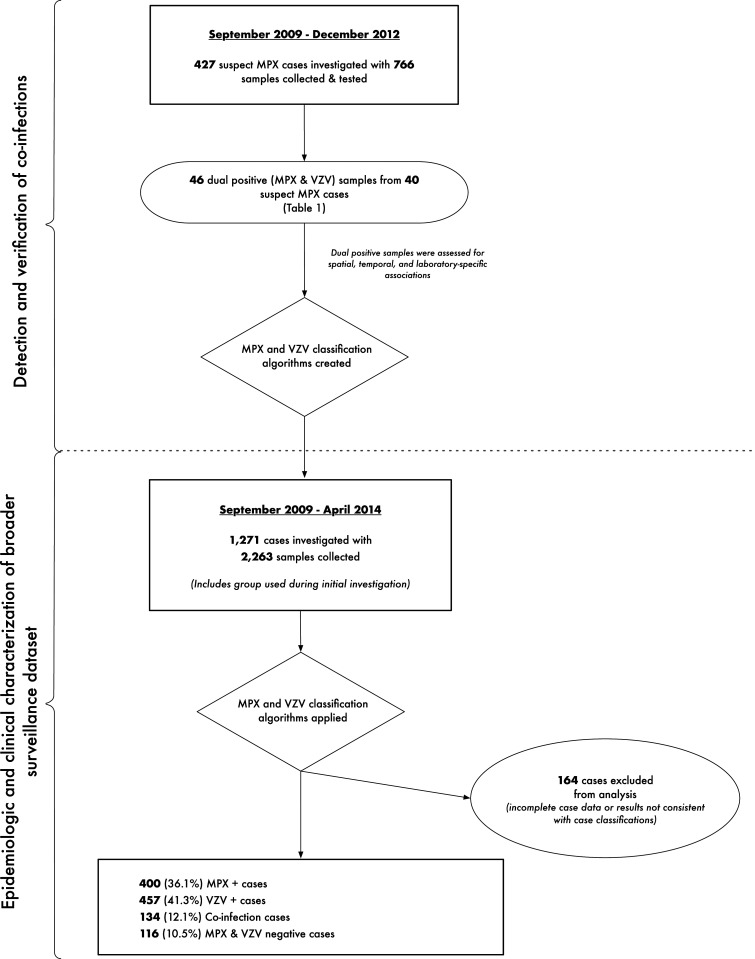
Flowchart for detection and verification of coinfections and subsequent characterization of the broader surveillance dataset for enhanced monkeypox (MPX) surveillance in Tshuapa Province, Democratic Republic of Congo.

Based on the initial analysis and laboratory results obtained for samples collected from suspected cases, a coinfection case classification algorithm was formulated. This algorithm was subsequently applied to the full body of surveillance data available for 1,271 suspected MPX cases, investigated between September 2009 and April 2014 (Figure 1). A total of 1,107 of the cases were categorized into four groups based on the classification algorithm: 1) MPX case, 2) VZV case, 3) MPX and VZV coinfection case, or 4) neither MPX nor VZV (i.e., a double-negative case). Demographic, clinical, and epidemiological data from case investigation forms were used for the analysis.

All statistical analyses were computed using SAS software version 9.3 (Cary, NC). A *P*-value < 0.05 was considered statistically significant. Frequencies were calculated for demographic data for each case category. Frequencies for reported signs and symptoms were calculated and stratified by case category. Time and space clustering of coinfection cases were assessed by evaluating health zone, illness onset, sample processing, and testing dates to look for associations. Comparisons of demographic characteristics and rates of signs and symptoms were made between case categories using chi-square or Fisher’s exact tests. Age was assessed as both a continuous variable (Student’s two-tailed *t*-test) and as a categorized variable (chi-square).

Rash burden was calculated using the sum of the reported lesion counts on each part of the patient’s body. The continuous variable for lesion count was compared between case categories using a pooled two-sample *t*-test. Cases were additionally categorized into rash burden groups established by the WHO: benign (1–25 lesions), moderate (26–100 lesions), grave (101–250 lesions), and plus grave (> 250 lesions). The distribution of rash burden groups was compared using a chi-squared test. Individuals were excluded from specific analyses when a variable of interest was missing from their case report forms.

## RESULTS

### Detection and verification of coinfections.

A large proportion of patients investigated between September 2009 (enhanced surveillance officially starting in 2010) and December 2012 were confirmed as both MPX and VZV cases based on results from independent MPX and VZV laboratory tests. Of the 427 cases (766 samples) investigated during this time, 40 (9.4%) were positive for both MPX and VZV.

The 40 individuals positive for both MPX and VZV had 57 samples (20 crust, 29 swab, and eight blood) processed and tested at the INRB, with their respective DNA remainders tested at the CDC. An additional 45 lesion samples from these individuals (21 crust and 24 swab) were processed and tested at the CDC. All 40 had at least one sample that was dual positive for MPX and VZV by PCR testing at the CDC. Fifty percent (*n* = 20) of these individuals had a dual positive crust sample, 45% (*n* = 18) had a dual positive swab sample, and 10% (*n* = 4) had a dual positive blood sample (Table 1). All dual positive swab and blood samples and 23 (48%) of dual positive crust samples tested at the CDC were DNA remainders from samples initially tested at the INRB. The remaining 12 dual positive crust samples were received at the CDC in their original form and were therefore able to be investigated further. The complete set of laboratory results for these 40 individuals is shown in Supplemental Table 1.

**Table 1:** CDC laboratory results for 40 coinfection cases with amplification of monkeypox and varicella zoster virus specific DNA sequences by PCR; samples collected from September 2009 to December 2012

Dual positive sample type	No of samples	Coinfection cases	
	*N*	*N*	%†
Crust	23	20	50
Swab	18*	18	45
Blood	5*	4	10

* All dual positive swab and blood samples were DNA remainders for samples processed at the Institut National de Recherche Biomédicale .

† Two individuals had more than one dual positive sample; therefore, percentages do not add to 100%.

**Figure 2.**
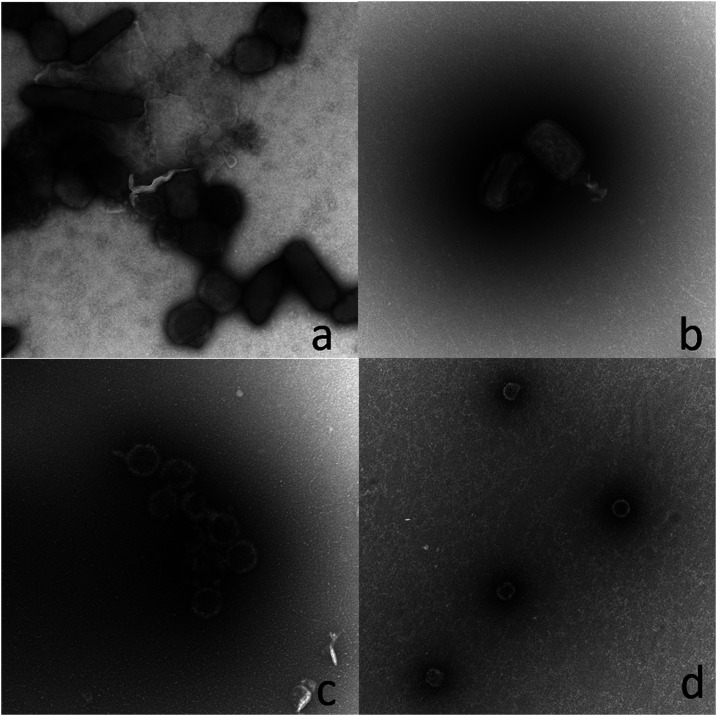
Electron micrographs showing *Orthopoxvirus* (OPX) and varicella zoster virus (VZV) virions from four dual positive crust samples. (**A** and **B**) OPX virions identified, no VZV virions (noted CPE in culture). (**C** and **D**) VZV virions identified, no OPX virions (no CPE in culture).

To better understand the biological plausibility of having two viruses present in a single skin lesion, the 12 dual positive crust samples were assessed for viable MPX and VZV via viral culture. Cytopathic effect consistent with OPX was observed in five of the 12 samples (38.5%). Negative stain EM was then performed on four of crust samples, two samples with and two without the noted cytopathic effect (Figure 2). In the two samples with cytopathic effect, OPX virions were detected by EM but no VZV virions. By contrast, VZV virions but no OPX virions were identified in the two samples that failed to propagate in culture. Viable MPX virus in these culture conditions resulted in cytopathic effect; therefore, the EM results for these four samples were consistent with their viral culture results.

A coinfection classification algorithm was created based on these findings. A patient was classified as a coinfection if 1) at least one sample was positive with OPX-generic assay or MPX-specific real-time PCR and 2) at least one lesion sample tested positive for VZV PCR testing at the CDC, or a lesion (crust) sample tested positive for VZV assay at the INRB. Varicella zoster virus-positive PCR results at the CDC from a DNA remainder crust sample were included as a confirmation for VZV when a primary lesion sample was not available for VZV testing.

### Epidemiologic and clinical characterization of broader surveillance data set.

One thousand two hundred seventy-one suspected MPX cases occurring between September 2009 and April 2014 were included in the final analysis. One thousand one hundred seven individuals had a sufficient, comprehensive set of laboratory results to be categorized using the case classification. Of these, 36.1% (400) were confirmed MPX cases, 41.3% (457) were confirmed VZV cases, 12.1% (134) were confirmed MPX/VZV coinfections, and 116 (10.6%) were negative for both MPX and VZV (Figure 1, Table 2). Males were a slight majority in all case categories. Although not significant, coinfection cases tended to be slightly younger (mean: 15.45 years) than MPX-alone (mean: 15.92 years) (*P* = 0.7) or VZV-alone cases (mean: 18.14 years) (*P* = 0.07).

**Table 2:** Age and gender of cases classified as MPX, VZV, or MPX/VZV coinfections; cases from surveillance dataset from September 2009 to April 2014

			Age (years) (continuous)		
Case classification	*n* (%)	% Male	Mean	Median	Range
MPX alone	400 (40.4)	52.9	15.9	13.8	(0.1–67.7)
VZV alone	457 (46.1)	52.9	18.2	13.0	(0.1–86.0)
Co-infections	134 (13.5)	51.1	15.5	11.0	(0.5–79.0)
Total cases	991 (100)	52.6	16.9	13.0	(0.1–86.0)

MPX = monkeypox; VZV = varicella zoster virus.

In addition to rash and fever, the most frequently reported signs and symptoms of coinfection cases included fatigue (86.1%), chills (80.1%), headache (73.9%), and myalgia (67.3%). When controlling for age, coinfection cases were significantly less likely to report signs/symptoms including mouth sores, axillary lymphadenopathy, cough, sore throat, and being “bed-ridden” than MPX cases (Table 3). Coinfection cases were significantly more likely to report signs/symptoms of fatigue, conjunctivitis, and being “bed-ridden” than VZV cases (Table 3).

**Table 3:** Significant differences in signs and symptoms for cases coinfected with MPX and VZV compared with patients with MPX or VZV alone, controlling for age.

Signs/symptom comparison*	Frequencies, %	Chi-square
	Coinfection vs. MPX	*P*-value
↓ Bed ridden	18.0 vs. 29.0	0.02
↓ Cough	46.2 vs. 58.6	0.02
↓ Mouth sores	41.5 vs. 58.7	0.001
↓ Sore throat	57.8 vs. 76.0	0.0002
↓ Axillary lymphadenopathy	43.6 vs. 58.2	0.006

	Coinfection vs. VZV	*P*-value
↑ Bed ridden	18.0 vs. 10.5	0.03
↑ Conjunctivitis	22.4 vs 11.8	0.004
↑ Fatigue	86.1 vs. 76.1	0.02

MPX = monkeypox; VZV = varicella zoster virus. Cases from surveillance dataset from September 2009 to April 2014. Based on 980 cases and classified as MPX, VZV, or MPX/VZV coinfections.

* Less than comparator (↓) greater than comparator (↑).

**Figure 3.**
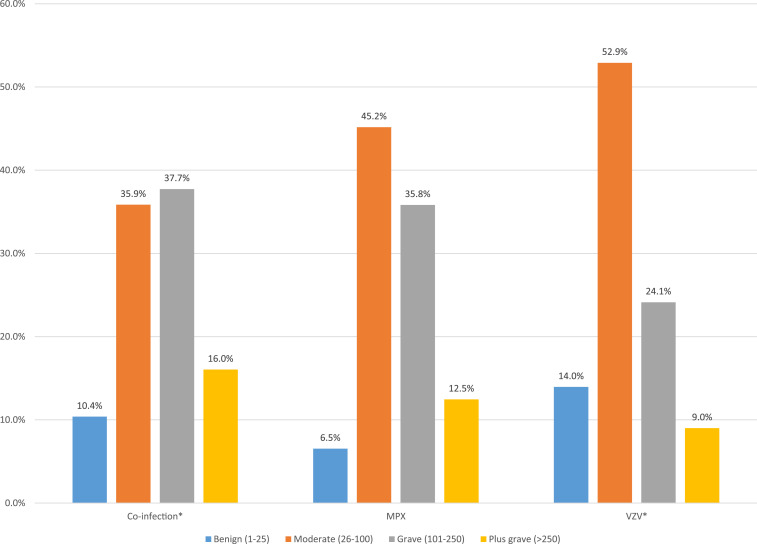
Rash burden classification among monkeypox (MPX), varicella zoster virus (VZV), and MPX/VZV coinfections. Rash burden is defined by the total sum of lesions on the body. *Coinfection cases were significantly more likely to be categorized into the higher rash burden categories than VZV cases (χ^2^ test, three degrees of freedom, *P* = 0.002).

**Figure 4.**
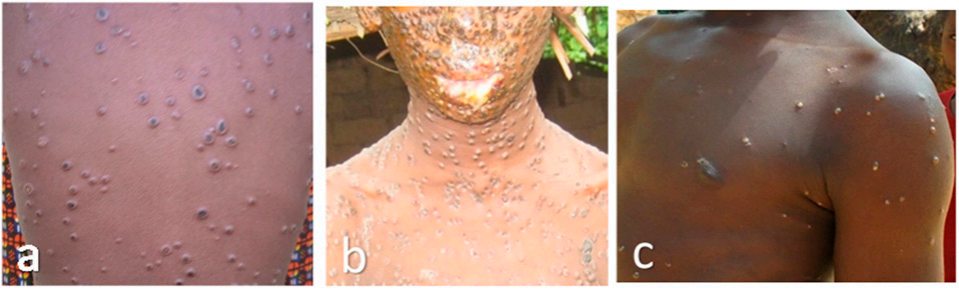
Rash distribution and burden examples for (**A**) coinfection case, (**B**) monkeypox-alone case, and (**C**) varicella zoster virus -alone case.

When comparing rash characteristics, coinfection cases were significantly less likely to have lesions on their face, thorax, arms, palms, and soles than MPX cases (chi-square *P*-values: 0.002–0.02). There were no significant differences in the presence of lesions by body location for coinfection cases compared with VZV cases. The total lesion count for coinfections (mean = 130, median = 104, range 3–431) was significantly higher than that for patients with VZV alone (mean = 104, median = 72, range 2–672; *P* = 0.025; Figure 3). Patients with MPX alone had the highest lesion counts (mean = 143, median = 98, range = 2–1,482); however, this was not significantly different from coinfection patients (*P* = 0.32). When categorized into rash burden groups, coinfections were significantly more likely to be categorized into the higher rash burden categories than VZV cases (*P* = 0.002; Figures 3 and 4).

Coinfection cases occurred in 10 of the 12 health zones in Tshuapa. The highest frequency of coinfection cases was observed in two adjacent zones, with one zone having 39 coinfections of 170 investigated (22.9%) and the other zone having nine coinfections of 57 investigated (15.8%). The zone with the largest number of coinfections (39 or 22.9% of investigated) also had the highest number of VZV-only cases (102/170 [60%]).

There were no discernible temporal or seasonal associations detected for coinfection cases, although the data available for these comparisons were somewhat limited. The proportion of coinfections among investigated cases remained constant month-to-month and year-to-year. There were no identified correlations between the timing of illness onset and sample processing for coinfection cases.

## DISCUSSION

Human MPX is a mandatory reportable disease in the DRC, and enhanced MPX surveillance was initiated in Tshuapa Province in 2010. In-depth case investigation forms allow for disease presentation and severity assessments. A unique feature available for this analysis was the collection of multiple lesion-derived samples from individual patients. In-country training has led to an increase in optimal sample type (swab/crust) collection for MPX laboratory-based diagnosis. Although previous studies have noted MPX and VZV coinfections in the DRC,^19^ this study was unique in that we were able to investigate biological plausibility of these coinfections by cell culture and EM analysis of dual positive original samples.

A large proportion of coinfections were unexpected at the initiation of this analysis; however, recent studies have shown similar rates of coinfections in the DRC.^19^ This evaluation of samples collected from coinfection cases in this study suggests that MPX and VZV cause distinct lesions. Not any sample from one lesion has shown to have both OPX and VZV virions by EM, although only a few lesion samples have been examined.

There were discernible differences found in the severity and reported symptoms for those with coinfections compared with those with either MPX or VZV alone. Coinfection cases were more likely to report signs/symptoms associated with MPX illness and had higher lesion burden than cases with VZV alone. However, coinfection cases were less likely to report these signs/symptoms and had an overall lower rash burden than cases with MPX alone. These findings indicate that coinfection with these two rash viruses could modulate the severity of the overall infection.

Co-circulation of both viruses,^25^ or an unknown pathogenic factor specific to infection with one or both viruses^18^ has been noted as an explanation for the coincidental detection of both viruses from a single patient. However, the mechanism behind the coinfection with MPX and VZV remains indefinite. One hypothesis is that initial infection with either virus causes the patient’s immune system to be more susceptible to a secondary infection. It is also possible that VZV lesions causing breaches in the skin may serve as an ideal entry point for MPXV infection after contact with infected individuals or animals. Recent serosurveys of young children in the DRC have shown that VZV seroprevalence is very low, and the comingling of these two viruses among a wide age-group is not unexpected.^16^

Another possibility is that infection with MPXV directly triggers VZV reactivation resulting in herpes zoster (HZ). Shedding of VZV from an index HZ case may result in transmission of the virus to susceptible persons in the community. Most coinfected individuals in this analysis were younger than would be expected for VZV reactivation; however, HZ can occur in younger individuals,^26^ and MPX might be able to trigger reactivation independently of age. Moreover, the ability of infectious agents or their components to induce the reactivation of herpesviruses from latency has been documented in the literature. Historic case reports following primary smallpox vaccination indicate infection with OPXs has been shown to activate HZ.^27,28^ Identifying and obtaining samples for PCR testing from the hypothetical index HZ case could be difficult, particularly if lesions have resolved before an outbreak is investigated. However, alternative diagnostic methods such as antibody avidity could help to elucidate this hypothesis. Underlying medical conditions (such as HIV) have not been well documented in this area and might also contribute to the risk of VZV reactivation.

### Limitations.

There were several limitations to this study. Education and training for enhanced surveillance in this area has dramatically improved the proportion of MPX cases among the total number of cases investigated. As a consequence, the proportion of VZV cases detected has decreased over the 4 years. The proportion of coinfection cases however has remained fairly consistent over this same time period. The lack of spatial or temporal associations for coinfections may be due to several factors. Fluctuations in the number of suspected cases investigated may be a by-product of outreach activities and weather, such as the rainy season. There may be a lag in reporting of suspected cases while surveillance officers are away from their posts, and subsequently an increase in cases once officers return for investigations. This could obscure any associations between case classifications and season/location. As enhanced surveillance continues, these short-term lags in investigations may decrease, allowing for a more robust analysis.

This analysis encompasses the early part of enhanced surveillance program (2010–2014). Increases in training and improvements in distinguishing MPX cases from VZV or other rash diseases are the primary goal, making it difficult to assess changes in incidence for these two viruses over time. Comparisons between coinfection cases and VZV-alone cases are also influenced by the MPX focus of the enhanced surveillance. Therefore, these VZV cases likely do not represent the full picture of VZV illness or incidence in this area.

Another limitation is the possibility for cross contamination between the multiple samples from one case. Results from this analysis indicate that coinfections have distinct MPX and VZV lesions. One possibility for crusts that were positive by PCR for both viruses but not by EM might be that multiple crusts are collected and placed into one tube, allowing for low levels of contamination that are detectable by a sensitive real-time PCR assay. Project personnel verified that independently collected crusts from a case were often placed together in a tube for transport to the laboratory. On the other hand, swabs are more likely to be collected and stored inside the individual swab sleeve, greatly reducing the chance for cross contamination. Cross contamination is unlikely to be a major factor in this study as five blood samples (four cases) were dual positive for MPX and VZV. Improving sample collection and storage procedures would allow for a better assessment of the viral makeup of individual lesions. Our sample set of double positive lesion samples was limited to make a more robust conclusion.

The co-circulation of similar diseases caused by distinct infectious agents is unusual if not unprecedented. During smallpox eradication, the disease most frequently mistaken for smallpox was varicella.^29^ Amid fears of bioterrorist deliberate release of smallpox, the CDC conducted a 2-year evaluation of a smallpox rule-out algorithm; 75% of cases suspected to be smallpox were confirmed as varicella infections.^30^ Since the implementation of universal childhood varicella vaccination in the United States, physicians have less experience with varicella and little experience with OPX infections and, in the context of lingering concerns about variola, may still mistake varicella for OPX disease. It will be important to pursue studies targeted at understanding mechanisms responsible for the presence of both MPX and VZV in rash illness outbreaks in Central Africa.

The collection of multiple lesion samples for this enhanced surveillance program allows for the detection of both MPX and VZV by PCR and the opportunity to detect coinfections. As this program continues, it will be important to continue these sample collection procedures to assess variations in the proportion of coinfected cases over time. In addition, an adjustment in the sample testing protocol in country may be prudent. If samples can be tested for both OPX/MPX and VZV at one time, it may help to provide a more comprehensive view of coinfection cases when combined with CDC laboratory results.

## Supplemental table

Supplemental materials
