# Supplementary material for: A Tale of Two Viruses: Coinfections of Monkeypox and Varicella Zoster Virus in the Democratic Republic of Congo
Source: Am J Trop Med Hyg. 2020 Dec 7;104(2):604–11. doi: 10.4269/ajtmh.20-0589 (PMC7866336; doi:10.4269/ajtmh.20-0589)
Supplement: Supplementary file 1 [file tpmd200589.SD1.docx]

The following are supplemental files and will be available online only

Supplementary Table 1. All laboratory results for initial set of 40 co-infections

| **Patient ID** | **Lab** | **Sample type*** | **Original sample type** | **MPX Specific PCR** | | **OPX PCR** | | **VZV PCR Result ^ǂ^** | **Culture results: Poxvirus** | **EM results** |
| --- | --- | --- | --- | --- | --- | --- | --- | --- | --- | --- |
|  |  |  |  | **Result** | **CT average** | **Result** | **CT average ƚ** |  |  |  |
| 1 | INRB | Crust | Crust | --- | --- | Negative | --- | Positive | --- | **---** |
| 1 | INRB | Swab | Swab | --- | --- | Negative | --- | Positive | --- | **---** |
| **1** | **CDC** | **Crust** | **Crust** | **Positive** | **34.90** | **Positive** | **30.87** | **Positive** | **---** | **---** |
| 1 | CDC | Swab | Swab | Negative | 40.00 |  |  | Positive |  | **---** |
| 2 | INRB | Crust | Crust | --- | --- | Positive | --- | Not Tested | --- | --- |
| 2 | INRB | Swab | Swab | --- | --- | Positive | --- | Not Tested | --- | --- |
| **2** | **CDC** | **Crust** | **Crust** | **Positive** | **20.10** | **---** | **---** | **Positive** | **---** | **---** |
| 2 | CDC | Swab | Swab | Positive | 17.83 | --- | --- | Negative | Positive | --- |
| 2 | CDC | DNA | Swab | Positive | 20.20 | --- | --- | Not Tested | --- | --- |
| 2 | CDC | DNA | Crust | Positive | 17.77 | --- | --- | Not Tested | --- | --- |
| 3 | INRB | Crust | Crust | --- | --- | Negative | --- | Positive | --- | **---** |
| 3 | INRB | Swab | Swab | --- | --- | Negative | --- | Positive | --- | **---** |
| **3** | **CDC** | **Crust** | **Crust** | **Positive** | **35.83** | **Positive** | **31.87** | **Positive** | **---** | **---** |
| 3 | CDC | Swab | Swab | Negative | 40.00 | --- | --- | Positive | --- | **---** |
| 4 | INRB | Blood | Blood | --- | --- | Negative | --- | Positive | --- | **---** |
| **4** | **CDC** | **DNA** | **Blood** | **Undetermined** | **39.17** | **Positive** | **36.66** | **Positive** | **---** | **---** |
| 5 | INRB | Swab | Swab | --- | --- | Not Done | --- | Not Tested | --- | --- |
| 5 | INRB | Blood | Blood | --- | --- | Negative | --- | Positive | --- | **---** |
| 5 | CDC | DNA | Swab | Undetermined | 39.70 | --- | --- | Positive | --- | **---** |
| **5** | **CDC** | **DNA** | **Blood** | **Positive** | **36.73** | **Positive** | **34.07** | **Positive** | **---** | **---** |
| 6 | INRB | Blood | Blood | --- | --- | Negative | --- | Positive | --- | **---** |
| 6 | INRB | Swab | Swab | --- | --- | Negative | --- | Positive | --- | **---** |
| **6** | **CDC** | **DNA** | **Swab** | **Undetermined** | **37.13** | **Positive** | **34.03** | **Positive** | **---** | **---** |
| 7 | INRB | Crust | Crust | --- | --- | Positive | --- | Not Tested | --- | --- |
| **7** | **CDC** | **Crust** | **Crust** | **Positive** | **18.87** | **---** | **---** | **Positive** | **Positive** | **---** |
| 7 | CDC | DNA | Crust | Positive | 24.73 | --- | --- | Not Tested | --- | --- |
| 8 | INRB | Swab | Swab | --- | --- | Negative | --- | Positive | --- | **---** |
| 8 | CDC | Swab | Swab | Negative | 40.00 | --- | --- | Positive |  | **---** |
| 8 | CDC | DNA | Swab | Negative | 40.00 | --- | --- | Positive |  | **---** |
| **8** | **CDC** | **Crust** | **Crust** | **Positive** | **24.10** | **---** | **---** | **Positive** | **Negative** | **---** |
| 9 | INRB | Crust | Crust | --- | --- | Negative | --- | Positive | --- | **---** |
| **9** | **CDC** | **Crust** | **Crust** | **Positive** | **26.20** | **---** | **---** | **Positive** | **Negative** | **---** |
| 9 | CDC | DNA | Crust | Undetermined | 39.80 | --- | --- | Positive | --- | **---** |
| 10 | INRB | Swab | Swab | --- | --- | Positive | --- | Not Tested | --- | --- |
| 10 | CDC | Swab | Swab | Positive | 19.50 | --- | --- | Negative | Negative | --- |
| **10** | **CDC** | **DNA** | **Swab** | **Positive** | **20.90** | **---** | **---** | **Positive** | **---** | **---** |
| 11 | INRB | Swab | Swab | --- | --- | Positive | --- | Not Tested | --- | --- |
| **11** | **CDC** | **DNA** | **Crust** | **Positive** | **22.40** | **---** | **---** | **Positive** | **---** | **---** |
| 12 | INRB | Crust | Crust | --- | --- | Positive | --- | Not Tested | --- | --- |
| 12 | INRB | Crust | Crust | --- | --- | Positive | --- | Not Tested | --- | --- |
| **12** | **CDC** | **Crust** | **Crust** | **Positive** | **16.30** | **---** | **---** | **Positive** | **Positive** | **---** |
| **12** | **CDC** | **DNA** | **Crust** | **Positive** | **16.70** | **---** | **---** | **Positive** | **---** | **---** |
| 13 | INRB | Swab | Swab | --- | --- | Positive | --- | Not Tested | --- | --- |
| 13 | INRB | Crust | Crust | --- | --- | Positive | --- | Not Tested | --- | --- |
| 13 | CDC | Swab | Swab | Positive | 19.80 | --- | --- | Negative | Positive | --- |
| 13 | CDC | Crust | Crust | Positive | 15.60 | --- | --- | Negative | --- | --- |
| **13** | **CDC** | **DNA** | **Crust** | **Positive** | **16.70** | **---** | **---** | **Positive** | **---** | **---** |
| 13 | CDC | DNA | Swab | Positive | 23.10 | --- | --- | Negative | --- | --- |
| 14 | INRB | Swab | Swab | --- | --- | Positive | --- | Not Tested | --- | --- |
| 14 | INRB | Crust | Crust | --- | --- | Positive | --- | Not Tested | --- | --- |
| 14 | CDC | Swab | Swab | Positive | 20.60 | --- | --- | Negative | --- | --- |
| **14** | **CDC** | **Crust** | **Crust** | **Positive** | **15.30** | **---** | **---** | **Positive** | **Positive** | **---** |
| 14 | CDC | DNA | Crust | Positive | 17.30 | --- | --- | Negative | --- | --- |
| 14 | CDC | DNA | Swab | Positive | 21.50 | --- | --- | Negative | --- | --- |
| 15 | INRB | Crust | Crust | --- | --- | Positive | --- | Not Tested | --- | --- |
| 15 | INRB | Swab | Swab | --- | --- | Positive | --- | Not Tested | --- | --- |
| **15** | **CDC** | **Crust** | **Crust** | **Positive** | **15.40** | **---** | **---** | **Positive** | **Positive** | **OPX virions detected; VZV virions not detected**  **(Figure 2 , Panel a)** |
| 15 | CDC | Swab | Swab | Positive | 19.10 | --- | --- | Negative | --- | --- |
| **15** | **CDC** | **DNA** | **Crust** | **Positive** | **18.10** | **---** | **---** | **Positive** | **---** | **---** |
| 15 | CDC | DNA | Swab | Positive | 20.70 | --- | --- | Negative | --- | --- |
| 16 | INRB | Swab | Swab | --- | --- | Positive | --- | Not Tested | --- | --- |
| 16 | CDC | Swab | Swab | Positive | 21.90 | --- | --- | Negative | Positive | --- |
| **16** | **CDC** | **DNA** | **Swab** | **Positive** | **21.50** | **---** | **---** | **Positive** | **---** | **---** |
| 16 | CDC | Crust | Crust | Negative | 40.00 | --- | --- | Positive | --- | **---** |
| 17 | INRB | Blood | Blood | --- | --- | Positive | --- | Not Tested | --- | --- |
| **17** | **CDC** | **DNA** | **Blood** | **Positive** | **34.00** | **---** | **---** | **Positive** | **---** | **---** |
| 18 | INRB | Blood | Blood | --- | --- | Positive | --- | Not Tested | --- | --- |
| **18** | **CDC** | **DNA** | **Blood** | **Positive** | **24.30** | **---** | **---** | **Positive** | **---** | **---** |
| 19 | INRB | Swab | Swab | --- | --- | Negative | --- | Positive | --- | **---** |
| 19 | INRB | Crust | Crust | --- | --- | Negative | --- | Positive | --- | **---** |
| 19 | CDC | Swab | Swab | Negative | 40.00 | --- | --- | Positive | --- | **---** |
| 19 | CDC | Crust | Crust | Negative | 40.00 | --- | --- | Positive | --- | **---** |
| 19 | CDC | DNA | Swab | Negative | 40.00 | --- | --- | Positive | --- | **---** |
| **19** | **CDC** | **DNA** | **Crust** | **Positive** | **36.10** | **---** | **---** | **Positive** | **---** | **---** |
| 20 | INRB | Crust | Crust | --- | --- | Positive | --- | Negative | --- | --- |
| 20 | INRB | Swab | Swab | --- | --- | Positive | --- | Not Tested | --- | --- |
| 20 | CDC | Crust | Crust | Negative | 40.00 | --- | --- | Positive | --- | **---** |
| 20 | CDC | Swab | Swab | Negative | 40.00 | --- | --- | Positive | --- | **---** |
| **20** | **CDC** | **DNA** | **Crust** | **Positive** | **34.70** | **---** | **---** | **Positive** | **---** | **---** |
| **20** | **CDC** | **DNA** | **Swab** | **Positive** | **35.90** | **---** | **---** | **Positive** | **---** | **---** |
| 21 | INRB | Crust | Crust | --- | --- | Positive | --- | Not Tested | --- | --- |
| **21** | **CDC** | **DNA** | **Crust** | **Positive** | **19.60** | **---** | **---** | **Positive** | **---** | **---** |
| 22 | INRB | Crust | Crust | --- | --- | Positive | --- | Not Tested | --- | --- |
| **22** | **CDC** | **DNA** | **Crust** | **Positive** | **20.90** | **---** | **---** | **Positive** | **---** | **---** |
| 23 | INRB | Swab | Swab | --- | --- | Positive | --- | Not Tested | --- | --- |
| **23** | **CDC** | **DNA** | **Swab** | **Positive** | **36.40** | **---** | **---** | **Positive** | **---** | **---** |
| 24 | INRB | Crust | Crust | --- | --- | Positive | --- | Not Tested | --- | --- |
| 24 | INRB | Swab | Swab | --- | --- | Positive | --- | Not Tested | --- | --- |
| **24** | **CDC** | **DNA** | **Swab** | **Positive** | **33.90** | **---** | **---** | **Positive** | **---** | **---** |
| 25 | INRB | Swab | Swab | --- | --- | Positive | --- | Not Tested | --- | --- |
| 25 | CDC | Swab | Swab | Positive | 21.60 | --- | --- | Negative | Negative | --- |
| **25** | **CDC** | **DNA** | **Swab** | **Positive** | **19.60** | **---** | **---** | **Positive** | **---** | **---** |
| 26 | INRB | Swab | Swab | --- | --- | Positive | --- | Not Tested | --- | --- |
| 26 | CDC | Swab | Swab | Negative | 40.00 | --- | --- | Negative | --- | --- |
| **26** | **CDC** | **DNA** | **Swab** | **Positive** | **36.50** | **---** | **---** | **Positive** | **---** | **---** |
| 27 | INRB | Swab | Swab | --- | --- | Positive | --- | Not Tested | --- | --- |
| 27 | CDC | Swab | Swab | Negative | 40.00 | --- | --- | Positive | --- | **---** |
| **27** | **CDC** | **DNA** | **Swab** | **Positive** | **34.90** | **---** | **---** | **Positive** | **---** | **---** |
| 28 | INRB | Swab | Swab | --- | --- | Positive | --- | Not Tested | --- | --- |
| 28 | CDC | Swab | Swab | Negative | 40.00 | --- | --- | Positive | --- | **---** |
| **28** | **CDC** | **DNA** | **Swab** | **Positive** | **35.90** | **---** | **---** | **Positive** | **---** | **---** |
| 29 | INRB | Crust | Crust | --- | --- | Negative | --- | Positive | --- | **---** |
| 29 | INRB | Swab | Swab | --- | --- | Negative | --- | Positive | --- | **---** |
| **29** | **CDC** | **Crust** | **Crust** | **Positive** | **28.40** | **---** | **---** | **Positive** | **Negative** | **OPX virions not detected, VZV virions detected**  **(Figure 2 , Panel d)** |
| 29 | CDC | Swab | Swab | Negative | 40.00 | --- | --- | Positive | --- | **---** |
| 29 | CDC | DNA | Crust | Negative | 40.00 | --- | --- | Positive | --- | **---** |
| 29 | CDC | DNA | Swab | Undetermined | 38.40 | --- | --- | Positive | --- | **---** |
| 30 | INRB | Crust | Crust | --- | --- | Positive | --- | Not Tested | --- | --- |
| 30 | CDC | Crust | Crust | Positive | 16.70 | --- | --- | Negative | Positive | --- |
| **30** | **CDC** | **DNA** | **Crust** | **Positive** | **21.50** | **---** | **---** | **Positive** | **---** | **---** |
| 30 | CDC | Swab | Swab | Negative | 40.00 | --- | --- | Positive | --- | **---** |
| 30 | CDC | DNA | Swab | Negative | 40.00 | --- | --- | Positive | --- | **---** |
| 31 | INRB | Swab | Swab | --- | --- | Positive | --- | Not Tested | --- | --- |
| 31 | CDC | Swab | Swab | Positive | 18.30 | --- | --- | Negative | Positive | --- |
| **31** | **CDC** | **DNA** | **Swab** | **Positive** | **22.20** | **---** | **---** | **Positive** | **---** | **---** |
| 32 | INRB | Swab | Swab | --- | --- | Positive | --- | Not Tested | --- | --- |
| 32 | CDC | Swab | Swab | Positive | 23.90 | --- | --- | Negative | Positive | --- |
| **32** | **CDC** | **DNA** | **Swab** | **Positive** | **22.00** | **---** | **---** | **Positive** | **---** | **---** |
| 33 | INRB | Crust | Crust | --- | --- | Positive | --- | Not Tested | --- | --- |
| 33 | CDC | Crust | Crust | Positive | 22.70 | --- | --- | Negative | Negative | --- |
| **33** | **CDC** | **DNA** | **Crust** | **Positive** | **26.50** | **---** | **---** | **Positive** | **---** | **---** |
| 34 | INRB | Blood | Blood | --- | --- | Positive | --- | Not Tested | --- | --- |
| 34 | INRB | Swab | Swab | --- | --- | Positive | --- | Not Tested | --- | --- |
| **34** | **CDC** | **DNA** | **Swab** | **Positive** | **23.00** | **---** | **---** | **Positive** | **---** | **---** |
| 34 | CDC | DNA | blood | Negative | 40.00 | --- | --- | Negative | --- | --- |
| 34 | CDC | Swab | Swab | Positive | 24.70 | --- | --- | Negative | Negative | --- |
| 35 | INRB | Blood | Blood | --- | --- | Positive | --- | Not Tested | --- | --- |
| **35** | **CDC** | **DNA** | **Blood** | **Positive** | **29.40** | **---** | **---** | **Positive** | **---** | **---** |
| 36 | INRB | Swab | Swab | --- | --- | Positive | --- | Not Tested | --- | --- |
| 36 | CDC | Swab | Swab | Positive | 20.40 | --- | --- | Negative | Positive | --- |
| **36** | **CDC** | **DNA** | **Swab** | **Positive** | **21.90** | **---** | **---** | **Positive** | **---** | **---** |
| 36 | CDC | Crust | Crust | Positive | 16.40 | --- | --- | Negative | --- | --- |
| 37 | INRB | Swab | Swab | --- | --- | Positive | --- | Not Tested | --- | --- |
| 37 | CDC | Swab | Swab | Positive | 21.60 | --- | --- | Negative | Negative | --- |
| **37** | **CDC** | **DNA** | **Swab** | **Positive** | **22.70** | **---** | **---** | **Positive** | **---** | **---** |
| 38 | INRB | Swab | Swab | --- | --- | Positive | --- | Not Tested | --- | --- |
| 38 | INRB | Crust | Crust | --- | --- | Positive | --- | Not Tested | --- | --- |
| 38 | CDC | Swab | Swab | Positive | 22.60 | --- | --- | Negative | Positive | --- |
| 38 | CDC | Crust | Crust | Positive | 16.70 | --- | --- | Negative | --- | --- |
| **38** | **CDC** | **DNA** | **Swab** | **Positive** | **22.40** | **---** | **---** | **Positive** | **---** | **---** |
| 38 | CDC | DNA | Crust | Positive | 19.40 | --- | --- | Negative | --- | --- |
| 39 | INRB | Swab | Swab | --- | --- | Negative | --- | Positive | --- | **---** |
| 39 | INRB | Crust | Crust | --- | --- | Negative | --- | Positive | --- | **---** |
| 39 | CDC | Swab | Swab | Negative | 40.00 | --- | --- | Positive | --- | **---** |
| **39** | **CDC** | **Crust** | **Crust** | **Positive** | **31.10** | **---** | **---** | **Positive** | **Negative** | **OPX virions not detected, VZV virions detected**  **(Figure 2 , Panel c)** |
| 40 | INRB | Swab | Swab | --- | --- | Positive | --- | Not Tested | --- | --- |
| 40 | INRB | Crust | Crust | --- | --- | Positive | --- | Not Tested | --- | --- |
| 40 | CDC | Swab | Swab | Positive | 19.30 | --- | --- | Negative | --- | --- |
| **40** | **CDC** | **Crust** | **Crust** | **Positive** | **16.10** | **---** | **---** | **Positive** | **Positive** | **OPX virions detected; VZV virions not detected**  **(Figure , Panel b)** |

**Bold font indicates single sample positive for MPX/OPX and VZV*** Sample type tested at PCR: "DNA" notation is for processed DNA remainders from INRB lab tested at CDC; "Crust" and "Swab" indicate original sample was tested
ƚ CT values for INRB OPX PCR results are not available
ǂ"Not Tested" for INRB lab VZV results are due to testing algorithm; "Not Tested" for CDC lab VZV results are due lack of sample remainder for testing
